# Antimelanoma potential of natural compounds derived from plants: a systematic review of *in vivo* studies of B16 melanoma and its sublines

**DOI:** 10.1590/1414-431X2025e14480

**Published:** 2025-10-06

**Authors:** B.S. Gomes, J.F.M. de Sousa, R.L. Mendes

**Affiliations:** 1 Programa de Pós-Graduação em Biociências, Universidade Federal do Vale do São Francisco, Petrolina, PE, Brasil; 2 Colegiado de Ciências Farmacêuticas, Universidade Federal do Vale do São Francisco, Petrolina, PE, Brasil

**Keywords:** Cutaneous melanoma, Medicinal plants, Antineoplastics, Natural products, Secondary metabolites

## Abstract

Cutaneous melanoma, a lethal neoplasm originating from epidermal melanocytes, stands out for its resistance to conventional therapies and high metastatic rate. Given the urgent need for new therapeutic approaches, this study focuses on the antitumor potential of natural compounds derived from plants, recognized as primary sources of antineoplastic chemotherapeutics. In this systematic review, we selected studies that evaluated the efficacy of such compounds *in vivo*, using the murine melanoma B16 model. The research was registered at the International Platform of Registered Systematic Review and Meta-Analysis Protocols under the number INPLASY202490019 and conducted using the PubMed, Science Direct, Scopus, and Embase databases, following predefined eligibility criteria and standardized terms from the MeSH and DeCS databases. The Preferred Reporting Items for Systematic Reviews and Meta-Analyses (PRISMA) guidelines were followed, and data were extracted using a Microsoft Excel^®^ spreadsheet. Of the 361 identified studies, 33 were deemed eligible for analysis. Parameters such as routes of treatment administration and induction of melanoma, duration of treatment, and others were collected. The SYRCLE tool was used to identify methodological gaps, such as the absence or insufficient description of randomization and blinding. The results indicated that natural products, especially terpenes, flavonoids, phenolic compounds, quinones, fatty acids, and plant sterols, have considerable antimelanoma activity, with tumor inhibition above 70% and antimetastatic properties. These findings underscore the importance of investigating the potential of these plant compounds as antineoplastic agents and establishing standardized experimental protocols to increase the reliability of the results.

## Introduction

Cutaneous melanoma originates from the autonomous proliferation of melanocytes, cells derived from the neural crest, which produce pigments (melanin) primarily located in the basal layer of the epidermis. Despite its low incidence compared to non-melanoma skin cancers, the lethality of this type of neoplasm is high. When diagnosed in the early stage of development, it presents a good prognosis. However, disease progression can lead to the formation of metastases, making it difficult to treat (1). Atypical melanocytes have the capacity to evade the homeostatic regulation of cellular growth, disseminate through tissues and organs, and withstand conventional chemotherapeutic treatments. Notably, tumor cells demonstrate the ability to keep intracellular drug concentrations low by expelling them into the extracellular space (2,3).

It is essential to develop new therapeutic alternatives that act to minimize the most aggressive biological behavior in advanced stages. The potential of natural products derived from plant species, which present numerous bioactive compounds, is highlighted. Scientific literature explains that more than 3000 plant species have antitumor activity and that about 85 of the 175 molecules approved by the Food and Drug Administration (FDA, USA) in the last 70 years come from natural products (4). Among such products used in medical clinics for melanoma treatment, vincristine and vinblastine, which are alkaloids isolated from *Catharanthus roseus*, stand out (4-7).

The antitumor activity of natural products derived from medicinal plants must be improved to better understand and explore the antineoplastic, antimetastatic, and antiangiogenic action of these plant species. An important tool for preclinical evaluation of these therapeutic agents is the murine melanoma model, particularly the one using the B16 cell line, derived from a spontaneous melanoma in C57BL/6 mice (8-10). The B16 model is widely recognized for its ability to replicate critical features of human melanoma, including rapid tumor growth and high metastatic potential, making it ideal for testing new therapeutic strategies such as plant-derived products (8-10). This review covers information on the experimental designs used to evaluate the antitumor activity *in vivo* and attempts to establish the effect of the tested secondary metabolites and their mechanisms of action in suppressing melanoma progression.

## Methodology

### Study type

This was a bibliographic data survey through a systematic review, presenting quantitative, exploratory, and descriptive methodological characteristics. This study was developed based on the following guiding question: “How effective are natural products derived from plant species in inhibiting the growth of murine melanoma when applied as treatment?”. This revision work was registered on the INPLASY (International Platform of Registered Systematic Review and Meta-Analysis Protocols) platform under number INPLASY202490019 (doi: 10.37766/inplasy2024.9.0019).

### Selection of databases and data search strategies

Data search was structured according to the guidelines provided by the Preferred Reporting Items for Systematic Reviews and Meta-analysis (PRISMA; Supplementary Tables S1 and S2). The databases used in this research were Science Direct, Scopus, PubMed, and Embase. The data search strategy employed standardized terms selected through vocabularies registered in MeSH (National Library of Medicine via Medical Subject Headings) and the Virtual Health Library using the Health Sciences Descriptors (DeCS). The terms used for data search were: “melanoma”, “medicinal plants”, and “antineoplastic agents”. The search method involved applying the terms to databases in a standardized way, using the Boolean operator “AND”, defining the following search strategy: “melanoma and (medicinal plants and antineoplastic agents)”. To conduct an updated systematic review of literature data, only studies published between 2013 and 2023 were included in the search, covering a period of 10 years (Supplementary Table S3).

### Study selection

The study selection was conducted independently by two reviewers (B.S. Gomes, J.F.M de Sousa) using the Rayyan platform - Intelligent Systematic Review. The selection process occurred in two stages: initially, reviewers screened the published studies by analyzing titles, abstracts, and identifying duplicates published in the different databases used in this research. Then, the reviewers assessed the inclusion of studies in this systematic review based on the eligibility criteria after reading the full articles, following predefined parameters. If any of the reviewers disagreed on the inclusion of a particular study, a third reviewer (R.L. Mendes) was consulted to provide a final opinion on whether or not the study should be included. To ensure consistency and reliability in the selection of studies and data extraction, the level of agreement between reviewers was analyzed using kappa coefficient (k), which quantifies the agreement while accounting for chance. A kappa value between 0.61 and 0.80 was considered indicative of substantial agreement in line with established guidelines (8).

The eligibility criteria were defined based on the PICOT strategy, ensuring methodological alignment and clarity. Studies were included if they used murine melanoma models, specifically the B16 lineage and its subline B16F10, to ensure consistency of the experimental approach. The interventions assessed involved the use of natural products from plant species, analyzed either in isolation or in combination, excluding pharmaceutical formulations or chemically synthesized compounds. As a comparison criterion, studies were required to include a negative control (inert substance) or a healthy group (Sham/Naive).

The evaluated outcomes were categorized into primary and secondary. The primary outcomes included antitumor and antimetastatic activity, with the investigated criteria being the tumor growth inhibition rate and tumor burden. The inhibition rate consists of the correlation between the average tumor weight of the treated group and the control group, and the tumor burden corresponds to the reduction in the number of metastatic nodules in the lungs, directly measuring the treatment's efficacy. The secondary outcomes included parameters related to complementary measures of tumor growth inhibition, such as tumor volume and weight, in addition to toxicological and tolerability aspects related to tumor progression and treatment response.

Additionally, only preclinical *in vivo* studies published between 2013 and 2023 in English, Portuguese, or Spanish were considered. Exclusion criteria were applied to ensure the relevance and quality of the selected studies. Studies that were not available in full text or presented only *in vitro* experimental designs were excluded. Research that used natural products without specifying the plant species of origin or exclusively analyzed natural products incorporated into pharmaceutical formulations were also excluded. Furthermore, publications categorized as systematic reviews, meta-analyses, case reports, clinical studies, conference articles, or other non-experimental publication types were not considered. The methodological quality of the studies was assessed using the SYRCLE tool, which identifies biases in animal model research. Criteria such as randomization, blinding, data completeness, and selective reporting were evaluated. This approach revealed methodological limitations and reinforced the reliability of the findings (11).

## Results and Discussion

During the screening phase of the studies, data search was conducted on electronic platforms, and 512 studies were identified, including 158 from Science Direct, 166 from Scopus, 100 from PubMed, and 88 from Embase. After excluding 57 duplicated studies, a total of 455 remained for further analysis.

**Figure 1:**
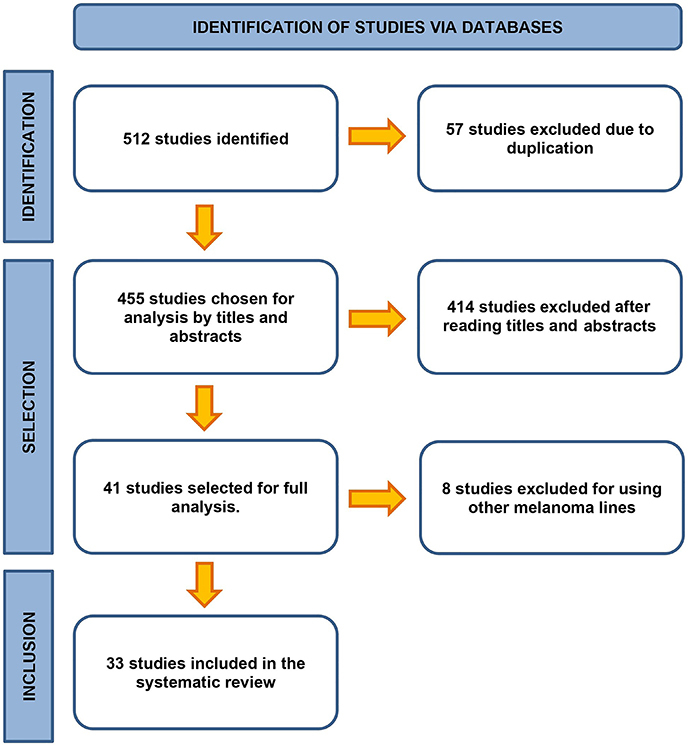
Flowchart of bibliographic search and study selection.

By examining the title and abstract of the studies, 414 articles that did not meet the inclusion criteria were excluded, leaving 41 studies eligible. Upon full-text review, 8 studies (Supplementary Table S4) were excluded as they used other melanoma cell lines in the *in vivo* tumorigenesis induction process, leaving 33 articles for the systematic literature review. The two reviewers agreed on the inclusion of the studies so that a third reviewer's evaluation was not needed. The flowchart in Figure 1 delineates the identification, selection, and inclusion phases. The kappa concordance coefficient was 0.7846, indicating a high level of concordance (8).

All studies were written in English. Among the selected works, there were 23 publications from Asian countries, with four from India (12-15), nine from China (16-24), four from South Korea (25-28), four from Tunisia (29-32), and two from Taiwan (33,34). Seven studies were from the Americas, with five from Brazil (35-39), one from Chile (40), and one from the United States (41). Some works were developed through partnerships between different countries, including one from Tunisia and France (42), one from Canada, Tunisia, and China (43), and one from China and the United States (44). Table 1 presents the general characteristics of the selected publications.

**Table 1:** General characteristics of the studies.

Study	Language	Country of origin	Plant species	Part of the plant	Natural product
Almeida et al. 2020 35	English	Brazil	*Athenaea velutina*	Leaves	Organic extract (Dichloromethane/Methanol)
Barros et al. 2020 36	English	Brazil	*Anacardium occidentale Linn*	Stem	Cashew gum - polymer
Bomfim et al. 2016 38	English	Brazil	*Annona vepretorum*	Leaves	Essential oil
Boubaker et al. 2018 29	English	Tunisia	*Nitraria retusa*	Leaves	Chloroform extract
Carvalho et al. 2013 37	English	Brazil	*Capraria biflora*	Roots	Biflorin
Chatti et al. 2022 30	English	Tunisia	*Rhamnus alaternus*	Leaves	Total oligomer flavonoid extract
Choudhari et al. 2023 14	English	India	*Prosopis juliflora*	Leaves	Methanolic extract
Dudek et al. 2015 41	English	United States	*Rhodiola crenulata*	Roots	Ethanolic extract
Fetter et al. 2021 39	English	Brazil	*Smilax fuminensis*	Leaves	Ethanolic extract
Gao et al. 2019 35	English	Taiwan	*Juniperus communis*	-	Extract
George; Kuttan 2016 13	English	India	*Emilia sanchifolia*	Complete plant	Standardized ethanolic extract
Hwang et al. 2013 25	English	South Korea	*Panax ginseng*	Roots	Gintonin
Kim et al. 2015 26	English	South Korea	*Angelica gigas Nakai*	-	Decursin
Kim et al. 2014 27	English	South Korea	*Illicium verum Hook*	Fruits	Aqueous extract
Krifa et al. 2014 31	English	Tunisia	*Limoniastrum guyonianum*	-	Aqueous extract
Krifa et al. 2016 32	English	Tunisia	*Pituranthos tortuosus*	Aerial parts	Essential oil
Li et al. 2013 16	English	China	*Xanthium strumarium*	Aerial parts	Xantathine
Li et al. 2017 24	English	China	*Sophora japonica/Lonicera japonica Thunb*	Flowers and flower buds	Ethanolic extract
Li et al. 2021 17	English	China	*Sophora japonica/Gardenia jasmimoides Ellis*	Flowers and flower buds	Ethanolic extract
Liu et al. 2019 18	English	China	*Sophora japonica/Lonicera japonica Thunb*	Flowers and flower buds	Ethanolic extract
Mohapatra et al. 2023 14	English	India	*Piper longum*	Fruits	Ethanolic extract
Mustapha et al. 2015 42	English	Tunisia/France	*Crataegus azarolus*	Leaves	Total oligomer flavonoid extract
Raja; Pandey 2020 15	English	India	*Lawsonia inermis*	Leaves	Methanolic extract
Robles-Planells et al. 2019 40	English	Chile	*Lithreae caustic*	Leaves	Ethereal extract
Sdiri et al. 2018 43	English	Canada/Tunisia/China	*Cynomorium coccineum*	Aerial parts	Aqueous extract
Son et al. 2014 28	English	South Korea	*Cynanchi atrati Radix*	-	Ethanolic extract
Wang et al. 2021 20	English	China	*Phaseolus vulgaris*	-	Phytohemagglutinin
Wang et al. 2022 21	English	China	*Astragalus propinquus Schischkin/Pinellia pedatisecta Schott*	Roots	Extract
Wang et al. 2022 22	English	China	*Garcinia hanburyi*	Resin	Gambogenic acid
Wang et al. 2023 19	English	China	*Paris polyphylla*	-	Polyphyllin B
Wu et al. 2013 44	English	China/United States	*Epimidium brevicormun/Epimidium sagittatum Maxim/Epimidium koreanum Nakai*	Aerial parts	Icariside II
Yu et al. 2017 34	English	Taiwan	*Solanum incanum*	-	Extract
Yu et al. 2023 23	English	China	*Vaccinium myrtillus L.*	Fruits	Bilberry anthocyanins extract

-: The study did not present the information.

The various derivatives of plant species resulted from different extraction methods, as well as separation, purification, and identification methods in the case of isolated substances. Of the natural products analyzed in the studies, 19 were organic extracts (12-15,17,18,21,23,24,28-30,33-35,39-42), three were aqueous extracts (27,31,43), two were essential oils (32,38), eight were isolated substances (16,19,20,22,25,26,37,44), and one was a polymer (36). The specifications of each evaluated natural product are outlined in Table 1.

The extraction method varies depending on the parts of the plants used to produce the derivative and the desired secondary metabolites. As shown in Table 1, nine studies used leaves (12,15,29,30,35,38-40,42), four used roots (21,25,37,41), four used aerial parts (16,32,43,44), three used flowers and floral buds (17,18,24), three used fruits (14,23,27), and one each used stems (36), the whole plant (13), and the resin (22), and seven did not specify the part of the plant used (19,20,26,28,31,33,34). Among the included publications, five analyzed natural derivatives resulting from the association of plant species (17,18,21,24,44). The application of more than one species to produce natural products is associated with ethnobotany, and the studies in question justified the choice of these derivatives based on Traditional Chinese Medicine and that they originated in China.

Animal models for melanoma studies are developed using different species of animals, including guinea pigs (*Cavia porcellus*), South American opossums (*Didelphis albiventris*), Syrian hamsters (*Syrian hamsters*), dogs (*Canis lupus familiaris*), goats (*Capra aegragus hircus*), fish (*Xiphophorus sp*.), and primarily mice (*Mus musculus*). Models employing mice have physiological, pathological, and histological similarities with human melanoma. Consequently, the most used experimental model involves the syngeneic transplantation of cells from the B16 lineage. Additionally, the C57BL/6 mouse strain is widely used, considering that the B16 cell line originates from a primary subcutaneous tumor that appeared spontaneously in a C57BL/6 mouse. This cell line was cultured through several passages, from which other strains with more aggressive features emerged, such as the B16F10 strain, which is highly metastatic (45). The experimental designs of *in vivo* models included the use of the C57BL/6 mouse strain in 26 studies and BALB/c in seven publications (12,29-32,39,42), as well as the use of the murine melanoma cell line B16 in tumor induction in four studies (34,40,43,44) and B16F10 in tumor induction in 29 publications, as shown in Supplementary Table S5.

Selecting the sex of mice increases the reliability of experiments by minimizing sex-related variability. Males were used in 21 studies and females in nine studies (14,23,27,33,35-37,41,44); three studies did not provide this information (12,40,43), as shown in Supplementary Table S5. Females are employed particularly due to their docile nature and easy handling. However, males are more frequently used because their hormonal fluctuations are milder (46).

The sample size (n) of animals varied between three and thirty per group, with five studies using n=5 (14,16,25,33,35), eight using n=6 (12,15,17-19,22,23,26), four using n=7 (20,24,33,42), five using n=8 (36,38,40,41,44), and four using n=10 (29,30,41,43). Some studies evaluated the antitumor and antimetastatic activity by distinct protocols (Supplementary Table S5). An appropriate and representative sample size is crucial for robust statistical analysis. Sample size calculation must consider that animals used in experiments are prone to frequent stress due to the induction of the lesion or disease model and treatment, leading to potential debilitation and eventual death. Additionally, adherence to the 3R principles (reduction, refinement, and replacement) is imperative, which aims to minimize the sample size, refine techniques, and replace *in vivo* analyses with alternative methods. This approach needs careful consideration of analysis requirements, the available experimental models, their similarity to human pathophysiology, and the desired outcomes (47,48).

The selected studies evaluated the antimelanoma potential of plant-derived compounds through protocols assessing either antitumor or antimetastatic activities, using different methods of tumor induction and parameters to verify the post-treatment outcomes. Typically, antitumor activity is assessed through the inhibition of tumor growth evaluated based on tumor volume. Conversely, antimetastatic activity is determined by counting the number of pulmonary metastatic nodules. Of the included studies, 24 conducted antitumor analysis (12,14-17,20-24,26,28-33,36,38-40,42-44), 6 focused on antimetastatic activity (13,18,27,35,37,41), and 3 evaluated both antitumor and antimetastatic effects (19,25,34) (Supplementary Table S5). The antitumor analysis of the study by Dudek et al. (41) was not included in this review because the natural product was administered topically. However, the antimetastatic assessment using the ethanolic extract of *Rhodiola crenulata* roots was included, as the plant derivative was used in its free form.

The treatments in the experimental protocols were administered through various routes. The intraperitoneal route was used in 21 studies (12,13,15,16,19,21,23,26,29-32,34-39,42-44), oral administration was used in eleven studies (14,15,17,18,21,23-25,27,28,41), the intratumoral route in two studies (34,42), and one study each was used the intramuscular (20) and subcutaneous (33) routes. The frequency of treatment in 21 studies was daily (12-14,16-18,21,23-25,27,28,30,32,34-40), while in eleven studies, it occurred on alternate days with a 48-h interval between administrations (15,19,21,26,29,31,33,41-44). In the study by Wang et al. (20), treatments were administered for five consecutive days followed by a 2-day break, then daily administration thereafter (Supplementary Table S5). The duration of treatments varied significantly, ranging from 10 to 40 days. However, the most common durations were 14 days (19,25,30,34) and 15 days (17,21,24,36), each observed in four studies, and 21 days (29,33,35,37,42) and 30 days (13,14,25,32,41) each used in five studies.

Thirty-two studies included a negative control group, except for Robles-Planells et al. (40), which utilized a healthy group for comparison. The negative control group consists of tumor-bearing animals treated with an inert substance, such as saline solution, phosphate-buffered saline (PBS), or the vehicle used for administering the natural product. Conversely, a positive control group, which received a clinically established active ingredient, was included in eleven studies (14-18,22,23,25,36,38,39). Additionally, a healthy group comprising healthy animals administered saline solution, PBS, or vehicle was featured in seven studies (14-16,29,31,40,42). Control groups play a pivotal role in the evaluation of the antitumor efficacy of the natural substances tested, facilitating the comparison of outcomes under these specific conditions, and substantiating the efficacy of the investigated products.

The parameters evaluated in the studies varied according to the type of analysis conducted. For antitumor analyses, the rate of tumor growth inhibition was used and for antimetastatic analysis, tumor burden, as indicated by the reduction in the number of metastatic nodules in the lungs, was used. However, twelve studies did not present this information in numerical form but rather through graphs, which precluded accurate data extraction (12,18,19,21-23,25,26,34,41,42,44).

**Figure 2:**
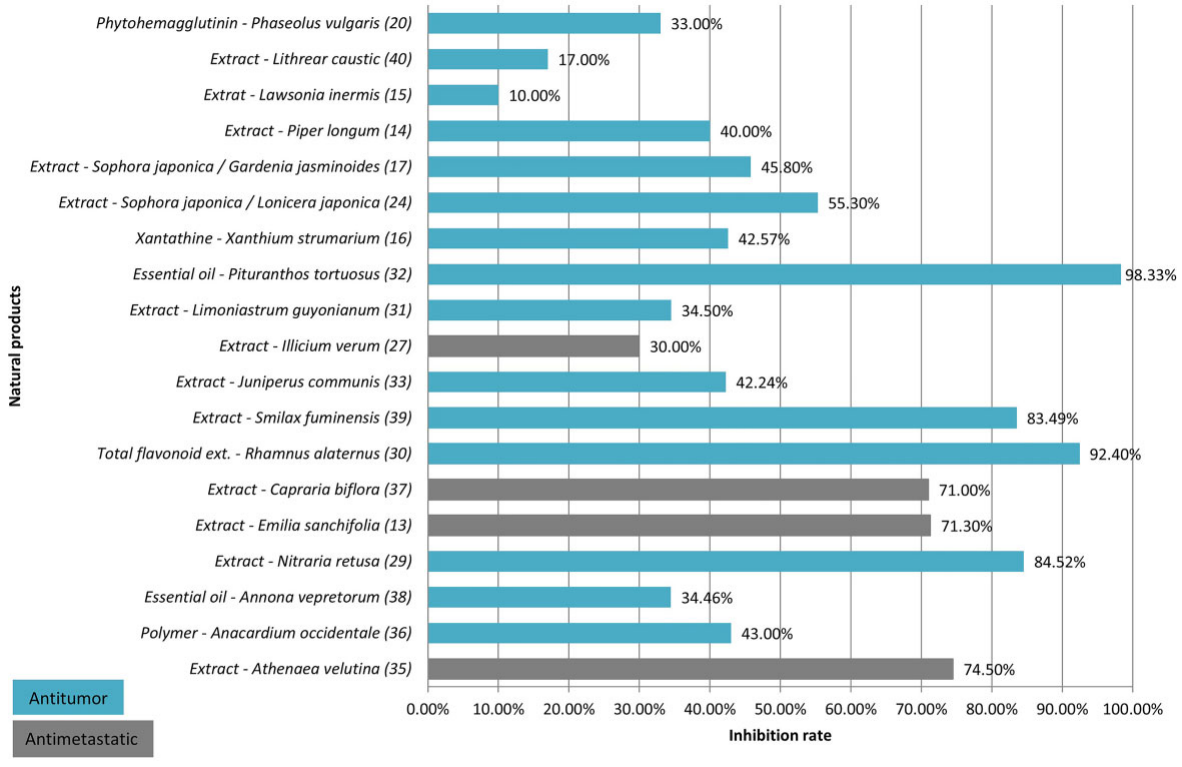
Percentage of tumor inhibition by treatments with medicinal plant derivatives.

Figure 2 and Table 2 show that the natural products inhibited melanoma by over 70%, with three studies (13,35,37) analyzing antimetastatic actions and four studies (29,30,32,39) focusing on antitumor effects. Among these, the essential oil extracted from the aerial parts of *Pituranthos tortuosus* demonstrated the most significant tumor growth inhibition, achieving 98.33% at a dosage of 100 mg/kg, as reported in the study by Krifa et al. (32). This species, endemic to North Africa, is known for its antimicrobial, antimutagenic, antifungal, cytotoxic, and immunomodulatory activities. Two of its secondary metabolites, terpinen-4-ol and D-limonene, have been found to contribute to its anti-melanoma activity. These compounds, classified as monoterpenes, are recognized for their active antitumor and antimetastatic properties, respectively.

**Table 2:** Experimental outcomes of the included studies.

Study	Concentration	Tumor weight	Tumor size	Inhibition rate	Secondary metabolite responsible for the action
		Secondary outcomes		Primary outcomes	
Almeida et al. 2020 35	100 mg/kg	131±10 number of pulmonary metastatic nodules	-	74.50%	Quinic acid derivatives; Flavonoids glycosides.
Barros et al. 2020 36	100 mg/kg	0.86±0.40 g	-	43.00%	Polysaccharides.
Bomfim et al. 2016 38	50 mg/kg	1.04±0.07 g	-	34.46%	Sesquiterpene; Monoterpene.
Boubaker et al. 2018 29	50 mg/kg	1.31±0.07 g	-	84.52%	β-sistosterol; Palmitic acid.
Carvalho et al. 2013 37	50 mg/kg	50±6 number of pulmonary metastatic nodules	-	71.00%	Quinone.
Chatti et al. 2022 30	100 mg/kg	0.11±0.02 g	129 mm^3^	92.40%	Flavonoids.
Choudhari et al. 2023 12	25 mg/kg	-	15 mm^3^	-	-
Dudek et al. 2015 41	100 mg/kg	0.30±0.05 g^a^	-	-	-
Fetter et al. 2021 39	100 mg/kg	0.07±0.02 g	88.1±35.4 mm^3^	83.49%	Phenolic compounds; Flavonoids.
Gao et al. 2019 33	200 mg/kg	-	861.84±245.44 mm^3^	42.24%	α-pinene; Citronellyl acetate; D-limonene (monoterpenes).
George; Kuttan 2016 13	25 mg/kg	71.62±19.30 number of pulmonary metastatic nodules	-	71.30%	Ƴ-humulene.
Hwang et al. 2013 25	100 mg/kg	1.8±0.7 number of pulmonary metastatic nodules	<1000 mm^3^	-	Gintonin (glycoprotein).
Kim et al. 2015 26	10 mg/kg	1.5±0.6 mg	2000±700 mm^3^	-	Decursin (coumarin).
Kim et al. 2014 27	50 mg/kg	402.6±160.6 number of pulmonary metastatic nodules	-	30.00%	Anethole.
Krifa et al. 2014 31	50 mg/kg	-	^-^	34.50%	Epigallocatechin (polyphenol).
Krifa et al. 2016 32	100 mg/kg	-	-	98.33%	Terpinen-4-ol; D-limonene.
Li et al. 2013 16	0.4 mg/10g	0.71±0.33 g	-	42.57%	Xanthatin (sesquiterpene lactone).
Li et al. 2017 24	1200 mg/kg	-	-	55.30%	Rutin; Luteolin; Quercetin.
Li et al. 2021 17	100 mg/kg	0.2±0.05 g^b^	-	45.80%	Crocine; Quercetin; Kampferol.
Liu et al. 2019 18	1200 mg/kg	5±2 number of pulmonary metastatic nodules	-	-	Rutin; Quercetin; Genistein.
Mohapatra et al. 2023 14	200 mg/kg	-	-	40.00%	Alkaloids; Lignans.
Mustapha et al. 2015 42	150 mg/kg	0.24 g	242 mm^3^	-	(−)-Epicatechin.
Raja; Pandey 2020 15	500 mg/kg	-	-	10.00%	-
Robles-Planells et al. 2019 40	0,1% (50 µL)	-	260 mm^3 e^	17.00%	Catechols; Sesquiterpenes.
Sdiri et al. 2018 43	50 mg/kg	-	-	****	-
Son et al. 2014 28	200 mg/kg	3.0 g	2800 mm^3^	75.00%^c^	Sibiricose A1; 1-O-b-D-glucopyranosyl sinapate; Sibiricose A4.
Wang et al. 2021 20	3 mg/mg	-	-	33.00%	Phytohemagglutinin (PHA-L).
Wang et al. 2022 21	25 + 50 mg/kg^d^	-	590 mm^3^	-	Hederaginina; Quercetina; β-sitosterol; Estigmastero.
Wang et al. 2022 22	8 mg/kg	-	-	-	Gambogenic acid.
Wang et al. 2023 19	1 mg/kg	-	-	-	Polyplhyllin B.
Wu et al. 2013 44	100 mg/kg	-	-	-	Icariside II (flavonol glycoside).
Yu et al. 2017 34	5 mg/kg	-	-	-	Solamargine.
Yu et al. 2023 23	45 mg/kg	4.0 g	-	-	Anthocyanins.

-: The study did not present information expressed numerically, reporting data using a graph without specifying the corresponding values; ^a^weight of the lung in the treated group, the organ affected by metastatic activity; ^b^SD: standard deviation; ^c^survival rate; ^d^ *Pinellia pedatisecta* Schott extract + *Astragalus propinquus* Schischkin extract; ^e^animals in the LExT group reached this tumor volume after 18-20 days; ****survival rate by Kaplan-Meier (range from 1 to 3 weeks).

The study conducted by Chatti et al. (30) assessed the total oligomeric flavonoid extract from the leaves of *Rhamnus alaternus* (100 mg/kg), which showed an inhibition rate of 92.40%. The likely mechanism of action for this derivative is linked to its capability to induce apoptosis and inhibit the adhesion, migration, and invasion of melanoma cells. Chemical analysis identified the presence of various flavones (quercetin diglucoside, quercetin-3-O-neohesperidoside, kaempferol-3-O-(2G-α-L-rhamnosyl)-rutinoside, rhamnetin hexoside, kaempferol-3-O-rutinoside, rhamnocitrin hexoside, pilosin hexoside, apigenin glucoside, and kaempferol-3-O-glucoside), which collectively contribute to the antimelanoma action.

The research by Boubaker et al. (29) documented a tumor growth inhibition rate of 84.52% with the chloroform extract of *Nitraria retusa* leaves (50 mg/kg). This effect is attributed to the constituents β-sitosterol and palmitic acid, which are thought to modulate various immunological effectors. The ethanolic extract of *Smilax fimunensis* leaves (100 mg/kg), investigated by Fetter et al. (39), resulted in a tumor inhibition rate of 83.49%. However, a concentration of 200 mg/kg exhibited an inhibition rate of 78.77%, suggesting that the antitumor potential does not display dose-dependent behavior. Additionally, histological studies showed more pronounced hepatic tissue alterations at the dose of 100 mg/kg given intraperitoneally. Phytochemical analysis revealed that the major compounds of the extract are flavonoids and phenolic compounds, both recognized for their antitumor activity, and the combination of these compounds is likely responsible for the mentioned antimelanoma activity.

The study by Almeida et al. (35) demonstrated a 74.50% reduction in the number of metastatic nodules in the lung through treatment with the organic extract of *Athenaea velutina* leaves (100 mg/kg), containing flavonoid glycosides and derivatives of quinic acid. Moreover, in the research by George and Kuttan (13), the standardized methanolic extract of *Emilia sanchifolia* (25 mg/kg) led to a 71.30% reduction in the number of metastatic nodules in the lung. This outcome can be associated with the main constituent of the extract (47.6%) γ-humulene (terpene), which has shown apoptotic effects in HT29 cells (human colorectal cancer) in previous studies. Lastly, treatment with the isolated compound biflorin (50 mg/kg) resulted in a 71.00% reduction in the number of metastatic nodules in the lung and increased the lifespan of the animals. This compound, isolated from the organic extract of *Capraria biflora* roots, is classified as a quinone. Its activity is attributed to alterations in protein profile and cellular invasiveness (37).

Seven studies exhibited the highest inhibition rates (>70%), and thus their experimental designs should be mentioned. All seven studies (13,29,30,32,35,37,39) utilized the B16F10 lineage or some strain variation for tumor induction; three studies (13,35,37) used C57BL/6 and four studies (29,30,32,39) used BALB/c. Additionally, two studies (35,37) used females and five studies (13,29,30,32,39) used males. The studies that exhibited inhibition rates closest to 100% (29,30,32,39) used males. This finding could be justified by the hormonal variation in females, which may interfere with tumor inhibition. Therefore, it is necessary to conduct studies to evaluate these potential alterations.

Six (13,29,30,32,37,39) of the seven studies with the highest tumor inhibition rates had samples (n) ≥10, with only the study carried out by Almeida et al. (35) using n=5. It is important to have a representative and reproducible sample for statistical analysis. Regarding treatment groups, only Fetter et al. (39) included a positive control group treated with active ingredients scientifically proven to be antineoplastics agents, serving as a comparison criterion. Incorporating this group increases the reliability of the results.

Of the seven studies (13,29,30,32,35,37,39) with the highest inhibition rates, only Boubaker et al. (29) administered treatments on alternate days with a 48-h interval between administrations. The other six studies administered treatments daily. Regarding the duration of the therapeutic intervention, all studies encompassed periods of more than 15 days, with two studies (13,32) lasting up to 30 days. The structure of the *in vivo* experimental design is crucial for obtaining reliable outcomes, as evidenced throughout this systematic review.

The application of the SYRCLE tool allowed for the identification of significant methodological gaps in the preclinical studies included. Among the evaluated criteria, randomization and blinding emerged as aspects frequently absent or insufficiently described, suggesting a potential increase in the risk of bias and compromising the internal validity of the results. On the other hand, aspects such as data completeness and the absence of selective outcome reporting were largely met, which increases the reliability in the findings. Additionally, the baseline characteristics of the experimental groups were generally well-balanced, suggesting that the groups were adequately comparable at the start of the experiments, which contributes to the robustness of the analyzed studies.

Based on these findings, it can be concluded that the evaluated natural products have significant antimelanoma activity. Despite the variety of the experimental designs, derivatives of medicinal plants exhibit relevant antitumoral and antimetastatic actions in the murine melanoma model, making them potential antitumor agents. Particularly noteworthy is the essential oil of *Pituranthos tortuosus*, which achieved a tumor growth inhibition of 98.33% when administered at a concentration of 100 mg/kg (32) and the total oligomeric flavonoid extract from *Rhamnus alaternus*, demonstrating a tumor inhibition rate of 92.40% at the concentration of 100 mg/kg (30). Therefore, this systematic review suggests that preclinical studies should continue to be developed to understand the action mechanisms of these plant-derived compounds and explore their safety and efficacy.

## Limitations

Some limitations were identified in the included studies through the application of the SYRCLE tool, which highlighted methodological gaps, such as the absence or insufficient description of randomization and blinding, which interferes with the comparability between experimental groups. Some studies did not present the tumor inhibition rate, a key metric for evaluating the antineoplastic potential of the tested substances. Instead, they reported qualitative data or complementary metrics, such as tumor volume or weight, making direct comparison between results difficult. Another notable issue was the lack of specificity regarding the part of the plant used to produce natural derivatives. Different plant organs have distinct chemical compositions, and the absence of this information hinders the identification of active metabolites and the analysis of potential mechanisms of action. Although aspects such as data completeness and the absence of selective reporting were largely met, ensuring greater reliability, the identified methodological gaps highlight the need for stricter planning and reporting in preclinical studies to enable the reproducibility and validity of the available evidence.

## Conclusions

The findings of this systematic review highlight the growing importance of natural products derived from plant species as potential therapeutic agents for melanoma treatment. The highest tumor growth inhibition rates were from compounds from various classes of secondary metabolites, including terpenes, flavonoids, phenolic compounds, quinones, fatty acids, and plant sterols. These diverse bioactive compounds underscore the untapped potential of phytochemicals in the development of innovative treatments for melanoma.

In conclusion, standardized, robust, and reproducible experimental designs are needed. These advancements will strengthen the evidence base, facilitate translational studies, and ultimately pave the way for the development of safe and effective natural product-based therapies for melanoma treatment.

## Supplementary Material

Click to view [pdf].
