# Supplementary material for: Antimelanoma potential of natural compounds derived from plants: a systematic review of in vivo studies of B16 melanoma and its sublines
Source: Braz J Med Biol Res. 2025 Oct 6;58:e14480. doi: 10.1590/1414-431X2025e14480 (PMC12513701; doi:10.1590/1414-431X2025e14480)
Supplement: Supplementary file 1 [file 1414-431X-bjmbr-58-e14480-suppl.pdf]

Table S1. PRISMA 2020 main checklist.

Topic	No.	Item	Location where item is reported
**TITLE**			
**Title**	1	Identify the report as a systematic review.	pp. 01
**ABSTRACT**			
**Abstract**	2	See the PRISMA 2020 for Abstracts checklist	
**INTRODUCTION**			
**Rationale**	3	Describe the rationale for the review in the context of existing knowledge.	pp. 01-02
**Objectives**	4	Provide an explicit statement of the objective(s) or question(s) the review addresses.	pp. 01-02
**METHODS**			
**Eligibility criteria**	5	Specify the inclusion and exclusion criteria for the review and how studies were grouped for the syntheses.	pp. 02
**Information sources**	6	Specify all databases, registers, websites, organisations, reference lists and other sources searched or consulted to identify studies. Specify the date when each source was last searched or consulted.	pp. 02
**Search strategy**	7	Present the full search strategies for all databases, registers and websites, including any filters and limits used.	pp. 02 Supplementary Table S3
**Selection process**	8	Specify the methods used to decide whether a study met the inclusion criteria of the review, including how many reviewers screened each record and each report retrieved, whether they worked independently, and if applicable, details of automation tools used in the process.	pp. 02
**Data collection process**	9	Specify the methods used to collect data from reports, including how many reviewers collected data from each report, whether they worked independently, any processes for obtaining or confirming data from study investigators, and if applicable, details of automation tools used in the process.	pp. 02
**Data items**	10a	List and define all outcomes for which data were sought. Specify whether all results that were compatible with each outcome domain in each study were sought (e.g. for all measures, time points, analyses), and if not, the methods used to decide which results to collect.	pp. 02
	10b	List and define all other variables for which data were sought (e.g. participant and intervention characteristics, funding sources). Describe any assumptions made about any missing or unclear information.	pp. 02
**Study risk of bias assessment**	11	Specify the methods used to assess risk of bias in the included studies, including details of the tool(s) used, how many reviewers assessed each study and whether they worked independently, and if applicable, details of automation tools used in the process.	pp. 02
**Effect measures**	12	Specify for each outcome the effect measure(s) (e.g. risk ratio, mean difference) used in the synthesis or presentation of results.	pp. 02
**Synthesis methods**	13a	Describe the processes used to decide which studies were eligible for each synthesis (e.g. tabulating the study intervention characteristics and comparing against the planned groups for each synthesis (item 5)).	pp. 02
	13b	Describe any methods required to prepare the data for presentation or synthesis, such as handling of missing summary statistics, or data conversions.	pp. 02
	13c	Describe any methods used to tabulate or visually display results of individual studies and syntheses.	pp. 02
	13d	Describe any methods used to synthesize results and provide a rationale for the choice(s). If meta-analysis was performed, describe the model(s), method(s) to identify the presence and extent of statistical heterogeneity, and software package(s) used.	pp. 02
	13e	Describe any methods used to explore possible causes of heterogeneity among study results (e.g. subgroup analysis, meta-regression).	pp. 02
	13f	Describe any sensitivity analyses conducted to assess robustness of the synthesized results.	pp. 02
**Reporting bias assessment**	14	Describe any methods used to assess risk of bias due to missing results in a synthesis (arising from reporting biases).	pp. 02
**Certainty assessment**	15	Describe any methods used to assess certainty (or confidence) in the body of evidence for an outcome.	pp. 02
**RESULTS**			
**Study selection**	16a	Describe the results of the search and selection process, from the number of records identified in the search to the number of studies included in the review, ideally using a flow diagram.	pp. 03
	16b	Cite studies that might appear to meet the inclusion criteria, but which were excluded, and explain why they were excluded.	pp. 03 Supplementary Table S4
**Study characteristics**	17	Cite each included study and present its characteristics.	pp. 03-08
**Risk of bias in studies**	18	Present assessments of risk of bias for each included study.	pp. 03-08
**Results of individual studies**	19	For all outcomes, present, for each study: (a) summary statistics for each group (where appropriate) and (b) an effect estimate and its precision (e.g. confidence/credible interval), ideally using structured tables or plots.	pp. 03-08
**Results of syntheses**	20a	For each synthesis, briefly summarise the characteristics and risk of bias among contributing studies.	pp. 03-08
	20b	Present results of all statistical syntheses conducted. If meta-analysis was done, present for each the summary estimate and its precision (e.g. confidence/credible interval) and measures of statistical heterogeneity. If comparing groups, describe the direction of the effect.	pp. 03-08
	20c	Present results of all investigations of possible causes of heterogeneity among study results.	pp. 03-08
	20d	Present results of all sensitivity analyses conducted to assess the robustness of the synthesized results.	pp. 03-08

Topic	No.	Item	Location where item is reported
**Reporting biases**	21	Present assessments of risk of bias due to missing results (arising from reporting biases) for each synthesis assessed.	pp. 08
**Certainty of evidence**	22	Present assessments of certainty (or confidence) in the body of evidence for each outcome assessed.	pp. 03-08
**DISCUSSION**			
**Discussion**	23a	Provide a general interpretation of the results in the context of other evidence.	pp. 03-08
	23b	Discuss any limitations of the evidence included in the review.	pp. 08
	23c	Discuss any limitations of the review processes used.	pp. 08
	23d	Discuss implications of the results for practice, policy, and future research.	pp. 03-09
**OTHER INFORMATION**			
**Registration and protocol**	24a	Provide registration information for the review, including register name and registration number, or state that the review was not registered.	pp. 02
	24b	Indicate where the review protocol can be accessed, or state that a protocol was not prepared.	pp. 02
	24c	Describe and explain any amendments to information provided at registration or in the protocol.	pp. 02
**Support**	25	Describe sources of financial or non-financial support for the review, and the role of the funders or sponsors in the review.	pp. 09
**Competing interests**	26	Declare any competing interests of review authors.	pp. 09
**Availability of data, code and other materials**	27	Report which of the following are publicly available and where they can be found: template data collection forms; data extracted from included studies; data used for all analyses; analytic code; any other materials used in the review.	Supplementary Material

Table S2. PRISMA abstract checklist.

Topic	No.	Item	Reported?
**TITLE**			
**Title**	1	Identify the report as a systematic review.	Yes
**BACKGROUND**			
**Objectives**	2	Provide an explicit statement of the main objective(s) or question(s) the review addresses.	Yes
**METHODS**			
**Eligibility criteria**	3	Specify the inclusion and exclusion criteria for the review.	Yes
**Information sources**	4	Specify the information sources (e.g. databases, registers) used to identify studies and the date when each was last searched.	Yes
**Risk of bias**	5	Specify the methods used to assess risk of bias in the included studies.	Yes
**Synthesis of results**	6	Specify the methods used to present and synthesize results.	Yes
**RESULTS**			
**Included studies**	7	Give the total number of included studies and participants and summarise relevant characteristics of studies.	Yes
**Synthesis of results**	8	Present results for main outcomes, preferably indicating the number of included studies and participants for each. If meta-analysis was done, report the summary estimate and confidence/credible interval. If comparing groups, indicate the direction of the effect (i.e. which group is favoured).	Yes
**DISCUSSION**			
**Limitations of evidence**	9	Provide a brief summary of the limitations of the evidence included in the review (e.g. study risk of bias, inconsistency and imprecision).	Yes
**Interpretation**	10	Provide a general interpretation of the results and important implications.	Yes
**OTHER**			
**Funding**	11	Specify the primary source of funding for the review.	Yes
**Registration**	12	Provide the register name and registration number.	Yes

From: Page MJ, McKenzie JE, Bossuyt PM, Boutron I, Hoffmann TC, Mulrow CD, et al. The PRISMA 2020 statement: an updated guideline for reporting systematic reviews. MetaArXiv. 2020, September 14. DOI: 10.31222/osf.io/v7gm2. For more information, visit: www.prisma-statement.org.

Table S3. Detailed search strategy.

Database	Search Terms	Boolean Operators Used	Fields Searched	Date Range	Language Restrictions
SCOPUS	(Melanoma) AND ((Plants Medicinal) OR (Plant Extracts)) AND (Antineoplastic Agents)	AND, OR	Title, Abstract, Keywords	2013-2023	English, Portuguese, Spanish
PubMed	(Melanoma) AND ((Plants Medicinal) OR (Plant Extracts)) AND (Antineoplastic Agents)	AND, OR	Title, Abstract, MeSH Terms	2013-2023	English, Portuguese, Spanish
ScienceDirect	(Melanoma) AND ((Plants Medicinal) OR (Plant Extracts)) AND (Antineoplastic Agents)	AND, OR	Title, Abstract, Keywords	2013-2023	English, Portuguese, Spanish
EMBASE	(Melanoma) AND ((Plants Medicinal) OR (Plant Extracts)) AND (Antineoplastic Agents)	AND, OR	Title, Abstract, Keywords	2013-2023	English, Portuguese, Spanish

Table S4. Studies Excluded for Using Other Cell Lines in Tumor Induction.

Study Title	Reason for Exclusion	DOI
Schisandrin B Inhibits Cell Viability and Malignant Progression of Melanoma Cells via Wnt/β-catenin Signaling Pathway	Used alternate melanoma cell lines not aligned with study focus	https://doi.org/10.2174/1871520623666230503094517
Discovery of potent immune-modulating molecule taccaoside A against cancers from structures-active relationships of natural steroidal saponins	The in vitro analyses were conducted with distinct cell lines and the in vivo studies were carried out with immunodeficient animals	https://doi.org/10.1016/j.phymed.2022.154335
Luteolin inhibits proliferation and induces apoptosis of human melanoma cells in vivo and in vitro by suppressing MMP-2 and MMP-9 through the PI3K/AKT pathway	Focused on different in vivo models than the study focus	https://doi.org/10.1039/C8FO02013B
Determination of Some Isoquinoline Alkaloids in Extracts Obtained from Selected Plants of the Ranunculaceae, Papaveraceae and Fumarioideae Families by Liquid Chromatography and In Vitro and In Vivo Investigations of Their Cytotoxic Activity	Different human melanoma cell lines were used and the in vivo was performed using a different xenograft model	https://doi.org/10.3390/molecules28083503
In vitro and in vivo anti-cancer effects of tillandsia recurvata (ball moss) from Jamaica	In vitro antiproliferation activity with different tumor cell lines including B16 melanoma and in vivo antitumor activity induced by Kaposi sarcoma	http://dx.doi.org/10.7727/wimj.2013.091
Evaluation of chemopreventive potentials of ethanolic extract of Ruta graveolens against A375 skin melanoma cells in vitro and induced skin cancer in mice in vivo	Chemopreventive study and in vivo analysis based on tumor induction by chemical agent	https://doi.org/10.1016/S2095-4964(15)60156-X
Extract of Cordyceps militaris inhibits angiogenesis and suppresses tumor growth of human malignant melanoma cells	In vivo assay tumor induction using the MeWo cell line	https://doi.org/10.3892/ijo.2014.2397
Anticancer effects of Ixeris dentata (Thunb. ex Thunb.) nakai extract on human melanoma cells A375P and A375SM	Melanoma induction through the association of A375P and A375SM cell lines	https://doi.org/10.1016/j.jep.2016.11.010

Table S5. Experimental designs of the included studies.

Study	Activity investigated	Cell line - route induction	Animal model	Sample size	Groups	Frequency - duration
Almeida et al. 2020 (35)	Antimetastatic	B16F10/intravenous	C57BL/6/female	n=5	Negative control; Treatment.	Daily/21 days
Barros et al. 2020 (36)	Antitumor	B16F10/subcutaneous	C57BL/6/female	n=8	Negative control; Positive control; Treatments.	Daily/15 days
Bomfim et al. 2016 (38)	Antitumor	B16F10/subcutaneous	C57BL/6/male	n=9/8	Negative control; Positive control; Treatments.	Daily/11 days
Boubaker et al. 2018 (29)	Antitumor	B16F10/subcutaneous	BALB/C/male	n=10	Healthy; Negative control; Treatment.	Every other day/21 days
Carvalho et al. 2013 (37)	Antimetastatic	B16F10/intravenous	C57BL/6/female	n=16	Negative control; Treatments.	Daily/21 days
Chatti et al. 2022 (30)	Antitumor	B16F10/subcutaneous	BALB/C/male	n=10	Negative control; Treatment.	Daily/14 days
Choudhari et al. 2023 (12)	Antitumor	B16F10/subcutaneous	BALB/C/–	n=6	Negative control; Treatment.	Daily/17 days
Dudek et al. 2015 (41)	Antimetastatic	B16F10/subcutaneous/ intravenous	C57BL/6/female	n=10/n=8	Negative control; Treatment.	Every other day/30 days
Fetter et al. 2021 (39)	Antitumor	B16F10/subcutaneous	BALB/C/male	n=11	Negative control; Positive control; Treatments.	Daily/17 days
Gao et al. 2019 (35)	Antitumor	B16F10/subcutaneous	C57BL/6/female	n=5/7	Negative control; Treatment.	Every two days/21 days
George; Kuttan 2016 (13)	Antimetastatic	B16F10/intravenous	C57BL/6/male	n=16	Negative control; Treatments.	Daily/30 days
Hwang et al. 2013 (25)	Antitumor/Antimetastatic	B16F10/subcutaneous/ intravenous	C57BL/6/male	n=5/n=5	Negative control; Positive control; Treatments.	Daily/30–14 days
Kim et al. 2015 (26)	Antitumor	B16F10/subcutaneous	C57BL/6/male	n=6	Negative control; Treatment.	Every other day/20 days
Kim et al. 2014 (27)	Antimetastatic	B16F10/intravenous	C57BL/6/female	n=4	Negative control; Treatment.	Daily/17 days
Krifa et al. 2014 (31)	Antitumor	B16F10/subcutaneous	BALB/C/male	n=30/15	Healthy; Negative control; Treatments;	Every other day/7, 14, 21 days
Krifa et al. 2016 (32)	Antitumor	B16F10/subcutaneous	BALB/C/male	n=15	Negative control; Treatment.	Daily/30 days
Li et al. 2013 (18)	Antitumor	B16F10/subcutaneous	C57BL/6/male	n=5/9	Healthy; Negative control; Positive control; Treatments.	Daily/13 days
Li et al. 2017 (24)	Antitumor	B16F10/subcutaneous	C57BL/6/male	n=7	Negative control; Treatment.	Daily/15 days

Li et al. 2021 (17)	Antitumor	B16F10/subcutaneous	C57BL/6/male	n=6	Negative control; Positive control; Treatment.	Daily/15 days
Liu et al. 2019 (18)	Antimetastatic	B16F10/intravenous	C57BL/6/male	n=6	Negative control; Positive control; Treatment.	Daily/16 days
Mohapatra et al. 2023 (14)	Antitumor	B16F10/subcutaneous	C57BL/6/female	n=5	Healthy; Negative control; Positive control; Treatments.	Daily/30 days
Mustapha et al. 2015 (42)	Antitumor	B16F10/subcutaneous	BALB/C/male	n=7	Healthy; Negative control; Treatment.	Every other day/21 days
Raja; Pandey 2020 (15)	Antitumor	B16F10/subcutaneous	C57BL/6/male	n=6	Healthy; Negative control; Positive control; Treatments.	Every other day/40 days
Robles-Planells et al. 2019 (40)	Antitumor	B16/subcutaneous	C57BL/6/–	n=8	Healthy; Treatment.	Daily/16 days
Sdiri et al. 2018 (43)	Antitumor	B16/intraperitoneal	C57BL/6/–	n=10	Negative control; Treatment.	Every other day/20 days
Son et al. 2014 (28)	Antitumor	B16F10/subcutaneous	C57BL/6N/male	n=20	Negative control; Treatments.	Daily/10 days
Wang et al. 2021 (20)	Antitumor	B16F10/subcutaneous	C57BL/6/male	n=7	Negative control; Treatments.	Diary with a two-day break/10 days
Wang et al. 2022 (21)	Antitumor	B16F10/subcutaneous	C57BL/6/male	n=4	Negative control; Treatments.	Daily/15 days
Wang et al. 2022 (22)	Antitumor	B16F10/subcutaneous	C57BL/6/male	n=6	Negative control; Positive control; Treatments.	Every other day/18 days
Wang et al. 2023 (19)	Antitumor/Anti metastatic	B16F10/subcutaneous/ intravenous	C57BL/6/male	n=6/n=3	Negative control; Treatments.	Every other day/14 days
Wu et al. 2013 (44)	Antitumor	B16/subcutaneous	C57BL/6/female	n=8	Negative control; Treatments.	Every other day/11 days
Yu et al. 2017 (34)	Antitumor/Anti metastatic	B16/subcutaneous/ intravenous	C57BL/6/male	n=4	Negative control; Treatment.	Daily/11–14 days
Yu et al. 2023 (23)	Antitumor	B16F10/subcutaneous	C57BL/6/female	n=6	Negative control; Positive control; Treatments.	Daily/17 days

–: The study did not present the information.
